# Lipocalin2 Induced by Bacterial Flagellin Protects Mice against Cyclophosphamide Mediated Neutropenic Sepsis

**DOI:** 10.3390/microorganisms8050646

**Published:** 2020-04-29

**Authors:** Daejin Lim, Hee Kyung Kim, Jae-Ho Jeong, Yoon Seok Jung, Shee Eun Lee, Hee-Chang Jang, Sook-In Jung, Hueng-Sik Choi, Joon Haeng Rhee, Sung-Gwon Lee, Chungoo Park, Miryoung Song, Hyon E. Choy

**Affiliations:** 1 Department of Microbiology, Chonnam National University Medical School, Gwangju 61468, Korea; kumdoman7@hanmail.net (D.L.); jeongjaeho@chonnam.ac.kr (J.-H.J.); 2 Department of Molecular Medicine (BK21plus), Chonnam National University Graduate School, Gwangju 61468, Korea; jhrhee@chonnam.ac.kr; 3 Department of Infectious Diseases, Chonnam National University Medical School, Gwangju 61648, Korea; hkthlove@hanmail.net (H.K.K.); haroc153@naver.com (H.-C.J.); sijung@chonnam.ac.kr (S.-I.J.); 4 National Creative Research Initiatives Center for Nuclear Receptor Signals, School of Biological Sciences and Technology, Chonnam National University, Gwangju 61186, Korea; yhemm@naver.com (Y.S.J.); hsc@chonnam.ac.kr (H.-S.C.); 5 Department of Pharmacology and Dental Therapeutics, School of Dentistry, Chonnam National University, Gwangju 61186, Korea; selee@chonnam.ac.kr; 6 Department of Microbiology and Clinical Vaccine R&D Center, Chonnam National University Medical School, Gwangju 61469, Korea; 7 School of Biological Sciences and Technology, Chonnam National University, Gwangju 61186, Korea; sunggwonl22@gmail.com (S.-G.L.); chungoo.park@gmail.com (C.P.); 8 Department of Bioscience and Biotechnology, Hankuk University of Foreign Studies, Yongin, Gyeonggido 17035, Korea

**Keywords:** chemotherapy, cytotoxicity, neutropenia, sepsis, lipocalin 2, *Enterobacteriaceae*

## Abstract

Neutropenic sepsis is a fatal consequence of chemotherapy, and septic complications are the principal cause of mortality. Chemotherapy-induced neutropenia leads to the formation of microscopic ulcers in the gastrointestinal epithelium that function as a portal of entry for intraluminal bacteria, which translocate across the intestinal mucosal barrier and gain access to systemic sites, causing septicemia. A cyclophosphamide-induced mouse model was developed to mimic the pathophysiologic sequence of events that occurs in patients with neutropenic sepsis. The TLR5 agonist bacterial flagellin derived from *Vibrio vulnificus* extended the survival of cyclophosphamide-treated mice by reducing the bacterial load in internal organs. The protective effect of flagellin was mediated by the antimicrobial protein lipocalin 2 (Lcn2), which is induced by TLR5-NF-κB activation in hepatocytes. Lcn2 sequestered iron from infecting bacteria, particularly siderophore enterobactin-dependent members of the *Enterobacteriaceae* family, thereby limiting their proliferation. Lcn2 should be considered for the treatment of neutropenic sepsis and gastrointestinal damage during chemotherapy to prevent or minimize the adverse effects of cancer chemotherapy.

## 1. Introduction

Chemotherapy can cause myelosuppression and fatally low levels of leukocytes, thereby increasing the susceptibility to infection and sepsis, which lead to increased morbidity and mortality in patients with malignancies. Cytoablative treatments cause marked degenerative changes in the small intestinal mucosa, resulting in villous atrophy and destruction of the lamina propria [1]. In addition, chemotherapy affects circulating immune cells and immune responses, which may cause immune suppression in cancer patients. The mechanisms underlying chemotherapy-induced immunosuppression include a decrease in lymphoid cells, disruption of tumor suppressing adenomatous polyposis coli function, increases in different subsets of myeloid cells, and upregulation of T cells [2]. Patients with malignancies eventually succumb to infection by intraluminal bacteria that translocate through the weakened intestinal mucosal barrier [3,4,5,6,7]. Possible approaches to reduce neutropenic sepsis-induced mortality include (i) rescuing depleted leukocytes, (ii) replenishing enterocytes in the gastrointestinal lining, and (iii) inhibiting growth of potentially dangerous bacteria.

Flagellin, a bacterial protein that polymerizes into the flagellar filament, is the only known agonist of toll-like receptor 5 (TLR5) that activates innate immunity, contributing to the immediate clearance of pathogens from the host [8,9,10,11,12]. We engineered a recombinant flagellin derived from *Salmonella* (CBLB502) that binds to TLR5 and specifically activates nuclear factor-kappa B (NF-κB), an essential mediator of immune responses. CBLB502 pretreatment protected mice against high-dose ionizing radiation by suppressing the induction of acute radiation syndromes involving the hematopoietic system and gastrointestinal tract [13]. In the gastrointestinal tract, CBLB502 pretreatment suppressed the radiation-induced decrease in small intestine crypt size and cell density by preserving normal levels of proliferative stem cells in the crypt and upregulated cytokines in the mouse plasma including radioprotective cytokines.

In this study, we examined the effect of a TLR5 agonist in a mouse model of cyclophosphamide (CPM)-induced neutropenic sepsis. CPM is a cytoablative agent that alkylates DNA to kill rapidly dividing cells [14,15] and is used to treat many types of cancer, including leukemia, myeloma, lymphoma, certain brain tumors, retinoblastoma, and prostate and breast carcinomas [16]. We used an engineered *Vibrio vulnificus* flagellin B (FlaB) that acts as a strong TLR5 agonist [17,18,19,20]. The results showed that the antimicrobial protein lipocalin 2 (Lcn2), which is induced by TLR5 signaling, had a protective effect on mice treated with CPM. Lcn2 (also known as neutrophil gelatinase-associated lipocalin (NGAL), siderocalin, or 24p3) is a member of the lipocalin superfamily and a pleiotropic mediator of various inflammatory processes [21,22]. Lcn2 is a bacteriostatic agent that interferes with siderophore (enterobactin)-mediated iron acquisition by various pathogenic bacteria in the family of *Enterobacteriaceae*. *Lcn*2^-/-^ mice are prone to infection and sepsis [23], suggesting that this pleotropic innate immune molecule promotes host resistance against infection. Consistently, in this study, Lcn2 had a protective effect on mice with CPM-induced enteritis.

## 2. Materials and Methods

### 2.1. Reagents, Flagellin, and Recombinant Lcn2 Peptide

CPM and the NF-κB inhibitor Bay11-7082 were purchased from Sigma-Aldrich (St. Louis, MO, USA). Mouse immortalized AML12 hepatocytes were obtained from ATCC Korea (ATCC CRL-2254). Engineered flagellin from *Vibrio vulnificus* (FlaB) was kindly provided by Dr. Shee Eun Lee (Chonnam National University Dental School, South Korea), and recombinant mouse Lcn2 (rmLcn2) was obtained from Sino Biological (Waynw, PA, USA).

### 2.2. Mouse Model

Eight-week-old male C57BL/6J mice were obtained from Samtako (Osan, South Korea). TLR5^−/−^ and *lcn2*^−/−^ mice were generously provided by Dr. Joon Haeng Rhee (Chonnam National University Medical School, South Korea) and Dr. Jang Soo Chun (Gwangju Institute of Science and Technology, South Korea), respectively. All mouse experiments were performed according to the guidelines of the Institutional Mouse Use and Care Commitment of Chonnam National University (CNU IACUC-H-2019-14). Neutropenic sepsis in mice was induced by intraperitoneal injection of CPM (500 mg/kg).

To evaluate the effect of flagellin, mice were pretreated with flagellin (15 μg/mouse) by intraperitoneal injection, and CPM was administered 30 min after flagellin pretreatment. The rmLcn2 was introduced into WT or *lcn2*^−/−^ mice intraperitoneally (100 μg/mouse) 24 h after CPM treatment. The survival time of each was measured, and survival rates were estimated using the Kaplan–Meier method.

### 2.3. Blood Leukocyte Count

Peripheral blood was collected from the retro-orbital venous sinus at 12, 24, and 72 h after CPM treatment and mixed 1:20 with Türk’s solution (YD Diagnostics, Yougin, South Korea) in a WBC pipette (Superior-Marienfeld, Lauda-Königshofen, Germany). Leukocytes were counted manually in each sample using the Neubauer Chamber (Brand GMBH, Wertheim, Germany) and by microscopic examination of gentian violet-stained samples.

### 2.4. Histopathological Analysis of the Intestines

Small and large intestines were collected from CPM-treated mice pretreated with or without flagellin at the indicated time points. The tissues were fixed in 10% neutral buffered formalin, embedded in paraffin, and sectioned at a thickness of 4 μm using a microtome (Leica Biosystems). After removal of paraffin, the sections were stained with hematoxylin and eosin for examination by optical microscopy (Leica Microsystems, CMS GmbH, Wetzlar, Germany).

### 2.5. Measurement of the Bacterial Burden in the Mouse Model

At the indicated time points (0, 12, 24, and 72 h) after CPM treatment, blood and organs including the liver, spleen, kidney, and lung were isolated from PBS or flagellin-pretreated WT mice. To determine the effect of rmLcn2, livers were harvested from WT or *lcn2*^−/−^ mice at 72 h after CPM treatment. All collected organs were homogenized in sterile PBS containing 0.05% Tween-20 using a Polytron homogenizer (IKA, Karnatnka, India). The homogenized organs were plated on 5% sheep blood agar plates (Synergy innovation, South Korea) and incubated at 37 °C for 24 h. The bacterial numbers were counted and the bacterial load was expressed as Colony Forming Units (CFUs)/g of each organ.

### 2.6. Sample Collection, DNA Isolation, and Sequencing

Liver and fresh fecal samples in the cecum were collected at 72 h after CPM or flagellin treatment and immediately frozen in liquid nitrogen before storage at −80 °C for further analysis. Genomic DNA from each sample was extracted using the FastDNA SPIN Kit for Soil (MP Biomedicals Inc., Irvine, CA, USA), according to the manufacturer’s instructions. To amplify the V3 to V4 regions of the 16S rRNA gene, PCR amplification was performed using the following primers: 341F (5′-TCGTCGGCAGCGTC-AGATGTGTATAAGAGACAG-CCTACGGGNGGCWGCAG-3′) and 805R (5′-GTCTCGTGGGCTCGG-AGATGTGTATAAGAGACAGGACTACHVGGGTATCTAATCC-3′). The primer above consisted of Nextera consensus, adapter and target regions separated by dashes. PCR amplification was performed under the following conditions: denaturation at 95 °C for 3 min; 25 cycles of 95 °C for 30 s, 55 °C for 30 s, and 72 °C for 30 s; and a final extension at 72 °C for 5 min. To attach the Illumina Nextera barcode, secondary amplification was performed with the i5 forward primer (5′-AATGATACGGCGACCACCGAGATCTACAC-XXXXXXXX-TCGTCGGCAGCGTC-3′, where X indicates the barcode region) and the i7 reverse primer (5′-CAAGCAGAAGACGGCATACGAGAT-XXXXXXXX-AGTCTCGTGGGCTCGG-3′). The conditions used were as mentioned above except that the amplification cycle was set to 8. To verify the size of PCR-enriched fragments, product size distribution was determined using the Agilent Technologies 2100 Bioanalyzer (Agilent Technologies, Palo Alto, CA, USA). The final purified products were quantified by qPCR using KAPA Library Quantification kits (KAPA Biosystems, St. Louis, MO, USA) for Illumina Sequencing platforms following the qPCR Quantification Protocol Guide. Purification of the PCR products and sequencing reactions were performed by ChunLab Inc. (Seoul, South Korea) with an Illumina MiSeq system (Illumina, San Diego, CA, USA).

### 2.7. Bioinformatics Analysis

After sequencing, raw reads were preprocessed using Trimmomatic (v0.32) with a mean phred score of 25 [24]. Next, primers of clean reads were trimmed using ChunLab’s in-house script with a similarity cutoff of 0.8. Nonspecific amplicons that did not encode a V3 to V4 region of the 16S rRNA gene were detected and removed using the HMMER hmmsearch program [25]. To minimize sequencing errors, amplicons were denoised using DUDE-Seq [26], and nonredundant reads were extracted using UCLUST-clustering [27]. Taxonomic assignment was performed using USEARCH [27] against the EzBioCloud database [28]. Taxonomic thresholds were determined according to Yarza et al. [29]. Chimeric sequences were discarded using the UCHIME software [30] and the EzBioCloud 16S rRNA database.

### 2.8. Measurement of Serum Lcn2 and Liver lcn2 Levels

Serum was isolated from collected blood samples by centrifugation. The total amount of Lcn2 was analyzed by ELISA using a mouse Lipocalin-2/NGAL Quantikine ELISA kit (R&D systems, Minneapolis, MN, USA) and SpectraFluor Plus (Tecan, Kanton Zürich, Swiss). Total RNA was isolated from the homogenized liver using TRIzol (Invitrogen, #15596018). The cDNA was synthesized by reverse transcription of the isolated RNA using the RT PreMix kit (Enzynomics, Daejeon, South Korea) and analyzed with the Rotor-Gene 6000 real-time PCR system (Qiagen, Hilden, Germany) using SYBR Green PCR Master Mix (Enzynomics, Daejeon, South Korea). The expression level of *lcn2* was normalized to the level of GAPDH. The primers used were as follows: lcn2 F (5′-GCAGGTGGTACGTTGTGGG-3′) and lcn2 R (5′-CTCTTGTAGCTCATAGATGGTGC-3′) for *lcn2*, and GAPDH F (5′- CCCACTAACATCAAATGGGG-3′) and GAPDH R (5′- CCTTCCACAATGCCAAAGTT-3′) for GAPDH.

### 2.9. Western Blot Analysis of Liver Lcn2

The livers were isolated from mice treated with flagellin by intraperitoneal injection. Each liver was homogenized in RIPA buffer (Thermo Fisher Scientific, Waltham, MA, USA), and the homogenized samples were centrifuged at 13,000 *g* at 4 °C for 1 h. The supernatant fraction containing extracted proteins (100 μg) was separated by 12% SDS-PAGE and transferred to PVDF membranes (Amersham, Buckinghamshire, England). Goat antimouse Lcn2 (R&D systems, Minneapolis, MN, USA) and mouse antimouse beta-actin (Santa Cruz, Dallas, TX, USA) were used as primary antibodies. Primary antibodies were diluted 1:1000 for Lcn2 or 1:3000 for beta-actin in TBS containing 0.2% Tween-20 (TBST) and incubated for 16 h at 4 °C. After washing with TBST, membranes were incubated with horseradish peroxidase-conjugated antigoat (Abcam, Cambridgeshire, England) or antimouse (ThermoFisher Scientific, Waltham, MA, USA) antibody in TBST for 1 h at room temperature. The signals were detected using chemiluminescence (Thermo Fisher Scientific, Waltham, MA, USA) and the BioRad chemidoc MP imaging system (Hercules, CA, USA).

### 2.10. Immunofluorescence Staining and Confocal Microscopy

After isolation, the livers of WT or *lcn2*^−/−^ mice were fixed in 4% paraformaldehyde, embedded in optimal cutting temperature compound (OCT; Tissue-Tek O.C.T. Compound, Wayne, PA, USA), and then frozen. The frozen livers were sliced into 6 μm-thick sections using a cryostat microtome (Leica Biosystems, Wetzlar, Germany), and the sections were mounted on aminopropyltriethoxysilane-coated slides. The slides were washed with PBS, pH 7.4, to remove the OCT, and incubated with 3% bovine serum albumin (*v/v* in PBS) for 1 h at room temperature for blocking. Then, the slides were incubated with goat antimouse Lcn2 antibody (R&D systems, Minneapolis, MN, USA) and rat antimouse F4/80 antibody (BioRad, Hercules, CA, USA) at 1:100 in PBS overnight at 4 °C. Alexa 594-conjugated donkey antigoat antibody (ThermoFisher Scientific, Waltham, MA, USA) and Alexa 488-conjugated goat antirat antibody (ThermoFisher Scientific, Waltham, MA, USA) were used as secondary antibodies diluted at 1:100 in PBS. The nuclei were stained with ProLong Gold antifade reagent with 4′,6-diamino-2-phenylindole (DAPI; ThermoFisher Scientific, Waltham, MA, USA). The fluorescent signals were imaged at a 200× magnification using a Zeiss confocal microscope (LSM 510, Zeiss Laboratories, Oberkochen, Germany). Representative images are shown.

### 2.11. Statistical Analysis

Data were analyzed using GraphPad Prism V7.0a software. The two-tailed Student’s *t*-test was used to estimate differences between two groups. Differences were considered statistically significant at *P* < 0.05.

## 3. Results

### 3.1. Protective Effect of a TLR5 Agonist (Bacterial Flagellin) on CPM-Treated Mice

**Figure 1:**
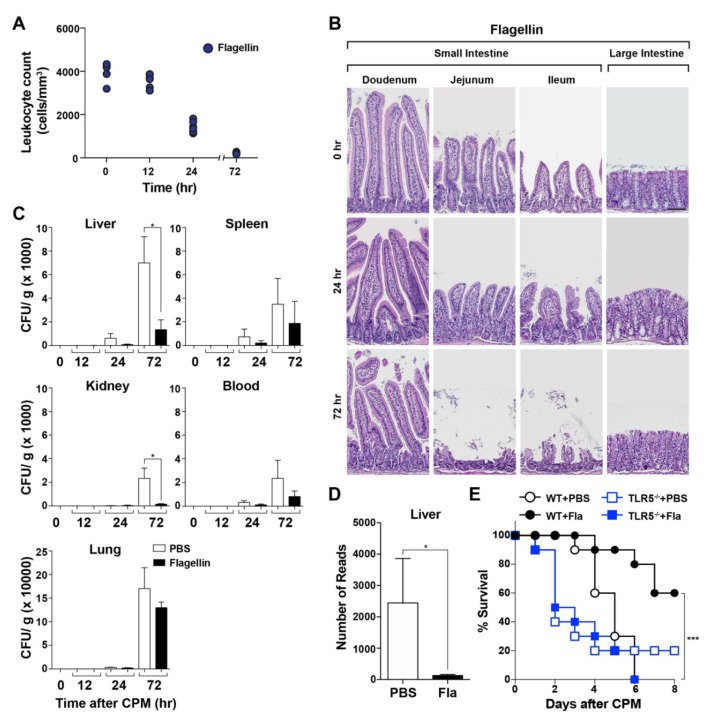
Effect of flagellin on cyclophosphamide (CPM)-treated mice. Thirty minutes prior to CPM-administration, mice were pretreated with flagellin (15 μg/mouse) through the intraperitoneal route on day 0. (**A**) Leukocytes were counted in each mouse at the indicated time points after CPM treatment by mixing the blood with Türk’s solution as described in Materials and Methods (*n* = 5 per group). (**B**) Representative images of hematoxylin and eosin (HE)-stained small and large intestines at the indicated times (h) after CPM treatment (*n* = 3). Scale bar, 100 μM. (**C**) The bacterial load was determined from the isolated liver, spleen, kidney, blood, and lung of CPM-treated mice with or without flagellin pretreatment (*n* = 5 per group) by plating on 5% sheep blood agar plates. Significance is indicated as * *P* < 0.05. (**D**) The number of 16S rRNA amplicons in mouse livers was determined as described above. Significance is indicated as * *P* < 0.05, *** *P* < 0.0008. (E) Survival of WT or TLR5^−/−^ mice treated with CPM in the absence (PBS) or presence of flagellin pretreatment (*n* = 10 per group).

To examine the protective effect of flagellin derived from *Vibrio vulnificus* on CPM-induced gastrointestinal toxicity, we developed and validated a polymicrobial sepsis model in the setting of CPM-induced neutropenia using C57BL/6 mice. Mice were pretreated with a strong TLR5 agonist, *Vibrio* flagellin, or PBS [17,18,19,20], 30 min before high-dose CPM injection (500 mg/kg; [7]). Leukocytes were counted at the indicated times (Figure 1A), and the gross morphology of the intestinal lining was examined (Figure 1B). The leukocyte count (cells/mm^3^) in the peripheral blood decreased to a similar extent in mice pretreated with *Vibrio* flagellin and in PBS-pretreated controls. On day 1, the leukocyte count decreased markedly to approximately 15% of the baseline (day 0, approximately 5000/mm^3^), further decreasing to <5% by day 3. This decrease reflects the susceptibility of neoplastic stem cells to the cytoablative effects of anticancer chemotherapy. These results demonstrated that CPM treatment induced near complete depletion of leukocytes in BL6 mice by day 3. Histological examination of the small intestinal mucosa revealed shortening of villi at 24 h and near complete destruction of villi and crypts at 72 h after CPM treatment in mice pretreated with *Vibrio* flagellin and in those treated with PBS (Figure 1B). Histopathological alterations were more pronounced in the small intestine than in the large intestine (colon), which was consistent with previous studies using different chemotherapeutic agents [31]. These results suggest that histological changes compromise the mucosal barrier function and increase intestinal permeability in both mice pretreated with PBS and in those pretreated with *Vibrio* flagellin [3,4,5,6,7]. Flagellin pretreatment did not preserve the gross morphology of the intestine in CPM-treated mice. Because CPM promotes the translocation of indigenous bacteria [32,33], we examined the bacterial load (colony forming units) in the liver, spleen, kidney, lung, and peripheral blood using cultured tissue extracts (Figure 1C) and in the liver by sequencing 16S rRNA gene (V3–V4 regions) bacterial amplicons (Figure 1D). The bacterial load was lower in the organs and serum of mice pretreated with flagellin than in those of PBS-pretreated mice at 72 h after CPM treatment (Figure 1C). The number of bacterial amplicons in flagellin-pretreated liver samples (average 128 reads) was approximately 5% of that in PBS-pretreated samples (average 2442 reads), suggesting that flagellin exerted antimicrobial effects (Figure 1D). Next, we examined the effect of flagellin on the survival of CPM-treated mice. Control mice treated with CPM became visibly ill after 2–3 days, and their condition became progressively worse until the mice reached a state of morbidity. A Kaplan–Meier survival plot indicated that the dose of CPM was high enough to kill all mice by day 6 starting on day 4 (Figure 1E). However, flagellin pretreatment extended the survival of CPM-treated mice by approximately 50%. To determine whether the protective effect was mediated by the interaction of flagellin with TLR5, the same test was performed in TLR5^−/−^ mice. TLR5^−/−^ mice were more sensitive to CPM treatment and no protective effect of flagellin was observed. These results suggest that an innate immune-related protein with antimicrobial effects induced by TLR5 signaling was responsible for the protective effect of flagellin on neutropenic septic mice.

### 3.2. Induction of Lcn2 by Flagellin in the Liver

**Figure 2:**
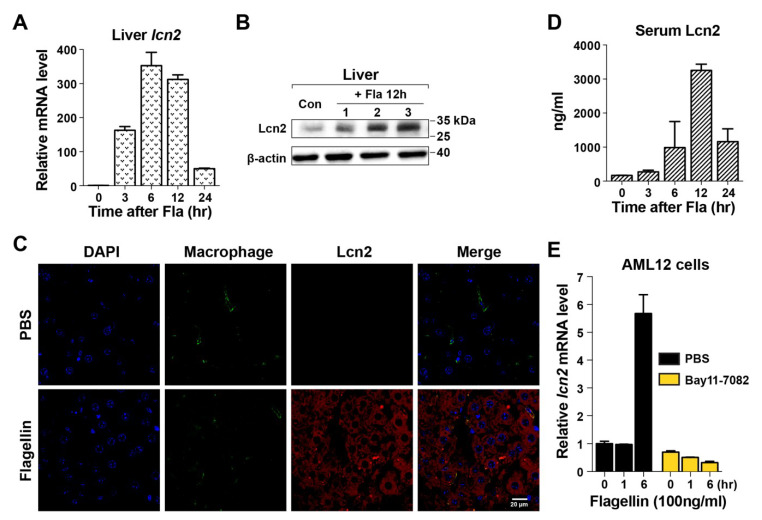
Induction of lipocalin 2 in hepatocytes by flagellin. (**A**) After flagellin treatment (15 μg/mouse), Lcn2 expression in the liver was measured by real-time PCR at the indicated time points (*n* = 5 for each experiment). The expression level was normalized to the mRNA levels of GAPDH in the same sample. The numbers shown for each time point are relative to the expression level at t = 0 in the untreated sample. Data are expressed as the mean ± SEM, and significance is indicated as * *P* < 0.01. (**B**) Lcn2 was detected in the liver of flagellin-treated mice (*n* = 3) after 12 h by Western blotting. Each lane represents an individual liver. PBS-treated samples were used as controls (first lane). (**C**) Histochemical analysis of Lcn2 in the liver of WT or *lcn2*^−/−^ mice at 12 h after flagellin treatment. PBS-treated mice were used as controls. Liver sections were costained with a macrophage marker, anti-F4/80 antibody (green), and DAPI (blue; nuclei). Lcn2 was detected using antimouse Lcn2 (red). Images were acquired at 400× magnification by confocal microscopy. (**D**) Lcn2 levels in mouse serum were measured by ELISA. (**E**) Mouse immortalized AML12 hepatocytes were treated with flagellin (100 ng/mL) together with PBS or the NF-κB inhibitor Bay11-7082. Lcn2 levels were determined by measuring Lcn2 mRNA expression relative to that of GAPDH by qPCR. The results were reproduced in three independent experiments.

Sepsis is accompanied by hypoferremia of inflammation, a primitive defensive mechanism to drastically reduce the concentration of circulating iron, thereby limiting its availability to pathogens [34,35]. Two host proteins are involved in the development of hypoferremia: hepcidin, which reduces the serum levels of iron, and Lcn2, which directly sequesters iron bound by the bacterial siderophore [34,35,36]. Both proteins are expressed in hepatocytes during bacterial infection. qPCR analysis showed that Lcn2 expression in the liver was 300-fold higher in mice treated with flagellin than in untreated mice (*t* = 0), and its levels peaked at 6–12 h after flagellin treatment (Figure 2A). This was verifed by Western blot analysis using a specific antibody at 12 h after treatment (Figure 2B). Histochemical analysis of the same samples detected Lcn2 exclusively in parenchymal hepatocytes (Figure 2C). Serum Lcn2 levels were significantly increased and peaked at 12 h after flagellin treatment (Figure 2D). The effect of flagellin on Lcn2 induction was examined in AML12 mouse immortalized hepatocytes, which showed that Lcn2 was induced at 6 h after flagellin treatment, whereas exposure to Bay11-7082, an NF-κB inhibitor, attenuated this effect (Figure 2E) [37]. Assessment of the expression of hepcidin in the liver of mice treated with flagellin showed that its induction was negligible compared with that of Lcn2 (Figure S1). These results indicate that Lcn2 expression in hepatocytes was induced by NF-κB signaling activated by the TLR5-flagellin interaction. This was confirmed by the absence of Lcn2 induction in primary hepatocytes derived from TLR5^−/−^ mice treated with flagellin (Figure S2).

### 3.3. Antimicrobial Effect of Lcn2 on CPM-Treated Mice

**Figure 3:**
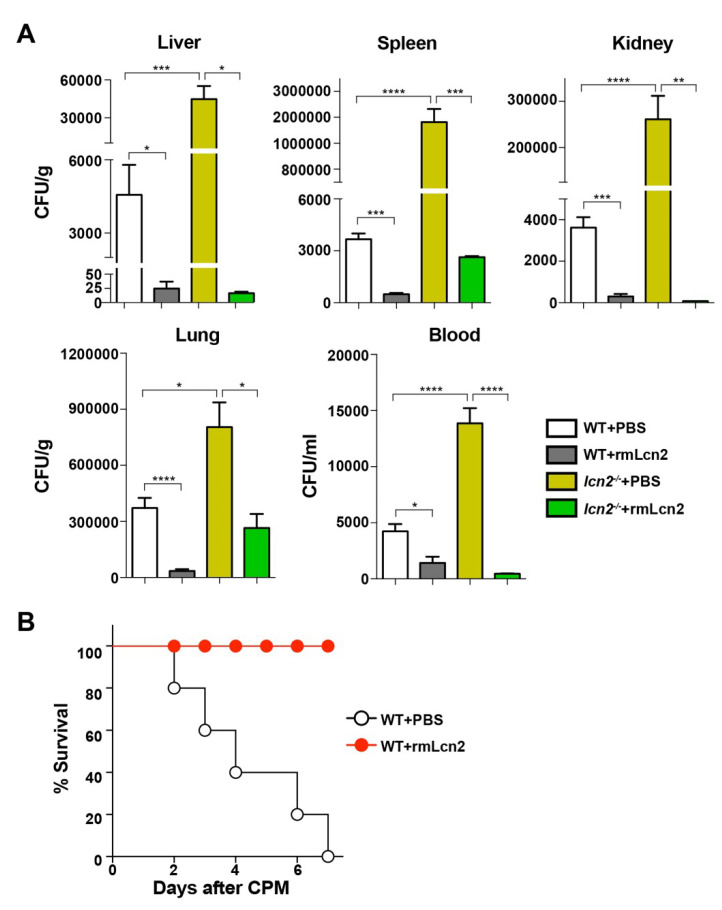
Effect of Lcn2 on neutropenic sepsis. Recombinant mouse Lcn2 (rmLcn2) was administered to mice once intraperitoneally (100 μg/mouse) at 12 h after CPM treatment. (**A**) At 72 h, bacterial numbers were counted in the liver, spleen, kidney, lung, and blood of CPM-treated mice injected with rmLcn2 or PBS (*n* = 5 per group). Data are expressed as the mean ± SEM, and significance is indicated as * *P*< 0.02, ** *P* < 0.005, *** *P* < 0.0008, and **** *P* < 0.0001. (**B**) Kaplan–Meier survival plot of WT mice treated with rmLcn2 or PBS at 12 h after CPM treatment (*n* = 5 per group).

To analyze the antimicrobial effect of Lcn2, mice were exposed to recombinant Lcn2 (mrLcn2) or PBS intraperitoneally at 12 h after CPM treatment. The bacterial load was measured in internal organs (liver, spleen, kidney, and lung) and in the blood of mice at 72 h after exposure to mrLcn2, which showed that mrLcn2 markedly decreased the bacterial load in the blood and in all organs tested (Figure 3A). Next, we examined whether mrLcn2 conferred a survival advantage to CPM-treated mice (Figure 3B). The protective effect of Lcn2 was comparable to that of flagellin (Figure 1). However, flagellin had no protective effect on the survival of *lcn2*^−/−^ mice (Figure S3). Taken together, these results indicate that Lcn2 is the antimicrobial effector protein induced by TLR5 signaling.

### 3.4. Effect of Lcn2 on the Enterobacteriaceae Family in a Mouse Model of Sepsis

**Figure 4:**
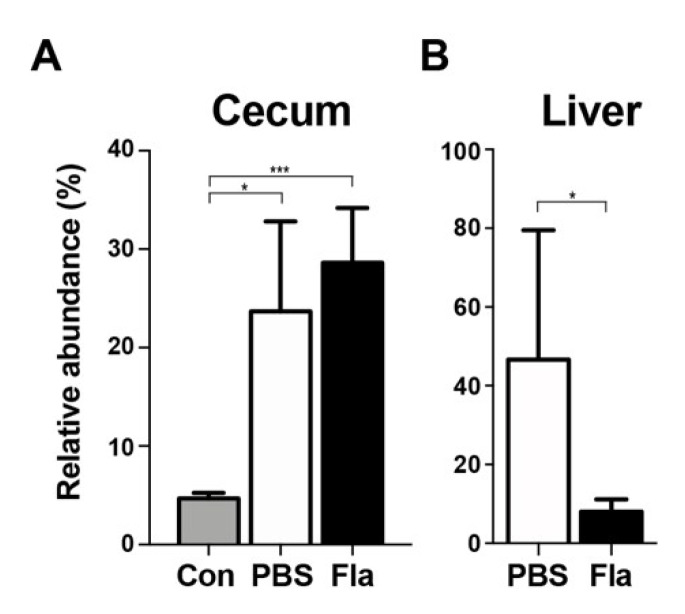
Relative abundance of *Enterobacteriaceae* in CPM-treated mice pretreated with flagellin. The 16S bacterial amplicons were determined in fresh fecal samples in the cecum (**A**) or the liver (**B**) at 72 h after CPM treatment. Mice were pretreated with PBS (*n* = 4) or flagellin (Fla; *n* = 5). Controls (Con; *n* = 3) were sham control animals without CPM treatment. Significance is indicated as * *P* < 0.02, *** *P* < 0.0008.

Lcn2 inhibits bacterial proliferation by sequestering the prototypical bacterial siderophore enterobactin, thereby inhibiting the growth of bacteria that rely on enterobactin for iron acquisition, namely, the members of the *Enterobacteriaceae* family [38]. The abundance of *Enterobacteriaceae* in the cecum and liver of CPM-treated mice was determined by sequencing 16S bacterial amplicons (Figure 4). The results showed that *Enterobacteriaceae* levels in the cecum were significantly higher in CPM-treated mice than in the controls, showing a 5-fold increase of 16S bacterial amplicons (despite minimal changes in the total number of amplicons (~50,000 reads). The 16S amplicons of *Enterobacteriaceae* in the liver of PBS-treated mice accounted for 46.675% of all bacterial amplicons, suggesting selective translocation of *Enterobacteriaceae* as shown previously in mice experiencing trauma [39,40]. Flagellin treatment decreased *Enterobacteriaceae* in the liver of mice to 7.8% of bacterial amplicons. Other bacterial phyla/families could not be measured because of the small number of 16S amplicons in the liver of flagellin-treated mice (average 128 reads, Figure 1D). These results suggest that Lcn2 induced by flagellin treatment selectively decreased the levels of enterobactin-dependent *Enterobacteriaceae.*

## 4. Discussion

This study demonstrated that the antimicrobial protein Lcn2 protects against CPM-induced cytotoxicity and bacterial sepsis and extends survival in an experimental model of polymicrobial sepsis designed to reproduce the pathophysiologic sequence of events that occurs following cytoablative chemotherapy (Figure 3). We showed that CPM treatment induced neutropenic sepsis in BL6 mice and demonstrated that Lcn2 extended the survival of neutropenic sepsis model mice. A study suggested that *Salmonella* flagellin, a TLR5 agonist, improves the therapeutic index of cancer radiotherapy [12]. This study suggested that *Salmonella* flagellin protected mice from the effects of high-dose ionizing radiation by inducing multiple plasma cytokines under the control of the NF-κB pathway, including radioprotective cytokines such as granulocyte colony-stimulating factor (G-CSF), interleukin 6, and tumor necrosis factor alpha [12]. In the present study, a similar protective effect was observed in neutropenic sepsis mice by *Vibrio* flagellin. Furthermore, we showed that the antimicrobial protein Lcn2 was involved in the protective effect of flagellin; administration of Lcn2 even at 12 h after CPM treatment conferred protection to neutropenic sepsis model mice (Figure 3). Lcn2 is produced by hepatocytes and neutrophils; extracellular Lcn2 secreted by hepatocytes limits systemic bacterial infection, whereas neutrophils carry the Lcn2 protein to local sites and protect against local bacterial infection [41]. Under the neutropenic sepsis conditions induced by CPM treatment, hepatocytes should be the sole source of Lcn2 production. Lcn2 expression is regulated by NF-κB [42]. The NF-κB consensus binding sequence 5′-GGGAATGTCC-3′ is present at the Lcn2 promoter sequence between −180 and −171 [43,44]. Coexpression of NF-κB and the IκB-ζ subunits synergistically activates the transcription of the Lcn2 gene in lipopolysaccharide-stimulated bone marrow-derived macrophages [45]. Consistently, the effect of flagellin on upregulating Lcn2 expression in hepatocytes in this study would be mediated by the activation of NF-κB signaling (Figure 2E) [43,44].

Upregulation of Lcn2 extended the survival of neutropenic sepsis mice, although an obvious effect on the preservation of the gastrointestinal lining or recovery of leukocyte counts was not observed (Figure 1). However, Lcn2 upregulation was associated with a significant reduction of the bacterial load in internal organs. In addition, administration of recombinant Lcn2 to neutropenic wild-type (WT) and *lcn2*^−/−^ mice extended survival by reducing the bacterial load (Figure 3). Lcn2 binds to enterobactin, which is produced and utilized by *Enterobacteriaceae* for iron acquisition, thereby inhibiting their growth [38,46]. It has been reported that *lcn2*^−/−^ mice have a 3-fold higher amount of fecal bacteria, most of which expresses *entA*, an enzyme involved in enterobactin synthesis, and harbors a large proportion of gram-negative bacteria (e.g., *Enterobacteriaceae*) in the gut [47]. We also observed a notable increase of *Enterobacteriaceae* in the cecum of CPM-treated mice, accounting for ~25% of the total number of bacterial amplicons (Figure 4). Trauma modifies the intestinal homeostatic environment, resulting in alterations in the intestinal microbiome and the overgrowth of *Enterobacteriaceae* [47,48], which is consistent with the present results. Translocation of *Enterobacteriaceae* to the mesenteric lymph nodes and eventual systemic sites would lead to sepsis and multiple organ failure. Consistently, 46.675% of bacterial amplicons in the liver of CPM-treated mice were *Enterobacteriaceae* family members. Flagellin treatment selectively reduced the population of septic *Enterobacteriaceae* to 7.8%, suggesting that Lcn2-mediated enterobactin sequestration delayed the growth of this family of bacteria (Figure 4).

**Figure 5:**
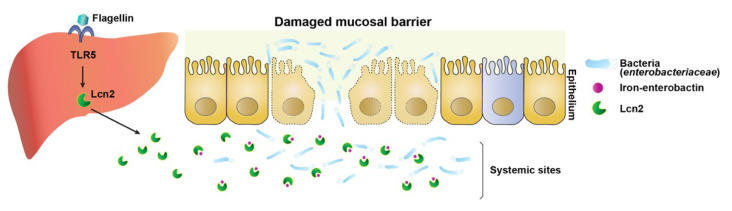
Graphical abstract. During neutropenic sepsis induced by cyclophosphamide treatment, intraluminal bacteria, particularly bacterial siderophore enterobactin-dependent *Enterobacteriaceae*, translocate to systemic sites through the deteriorated intestinal barrier and proliferate using available iron. Lipocalin 2, induced by activation of TLR5-NF-κB in hepatocytes by Vibrio flagellin, chelates the iron, creating an iron limiting condition that inhibits the growth of *Enterobacteriaceae*.

There are various strategies for preventing neutropenic sepsis. Prophylactic strategies include administration of G-CSF or antibiotics and altering the cytotoxic regimen. The use of G-CSF to prevent neutropenic sepsis has substantially improved patient survival [49]. G-CSF is a colony-stimulating hormone that increases the neutrophil count, shortening the duration of neutropenia by stimulating neutrophil production by the bone marrow. However, adverse effects include bone pain, headache, and nausea, and rarely more serious complications such as anaphylaxis, respiratory failure, and splenic rupture. Prophylactic use of antibacterial agents during the early afebrile period can reduce the frequency of febrile episodes [50,51,52]. However, antibiotic prophylaxis may encourage the growth of new and more resistant infective agents. In this study, we demonstrate for the first time that Lcn2 effectively retards the outgrowth of the *Enterobacteriaceae* family at systemic sites to which it translocates through the injured gastrointestinal lining from disturbed intraluminal sites (Figure 5). Our data indicates that Lcn2 could be an important player in the treatment of neutropenic sepsis (Figure 3). 

## Supplementary Materials

The following are available online at https://www.mdpi.com/2076-2607/8/5/646/s1.
