# Supplementary material for: Lipocalin2 Induced by Bacterial Flagellin Protects Mice against Cyclophosphamide Mediated Neutropenic Sepsis
Source: Microorganisms. 2020 Apr 29;8(5):646. doi: 10.3390/microorganisms8050646 (PMC7284693; doi:10.3390/microorganisms8050646)
Supplement: Supplementary file 1 [file microorganisms-08-00646-s001.pdf]

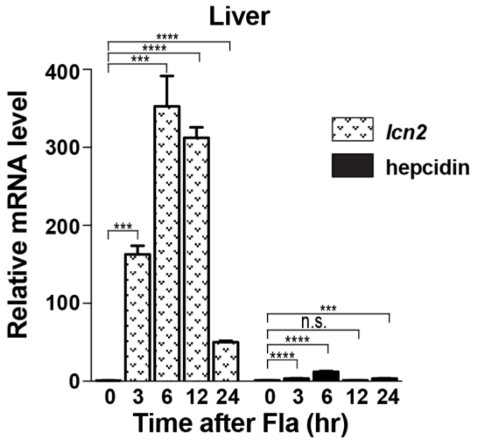

Supplemental Figure 1. Hepcidin expression in the livers of mice treated with flagellin. Mice were treated with flagellin (15 μg/mouse) through the intraperitoneal route on day 0. Hepcidin expression in the liver was measured by real-time PCR at the indicated time points (n = 4 for each experiment) and normalized to GAPDH mRNA expression in the same samples. Numbers at each time point represent mRNA expression relative to that at t = 0 in untreated samples. Data are expressed as the mean ± SEM, and significance is indicated as *** $P < 0.0005$ and **** $P < 0.0001$. To simplify the comparison, hepcidin expression levels at the indicated times are displayed together with Lcn2 levels in the same liver samples from Figure 2B.

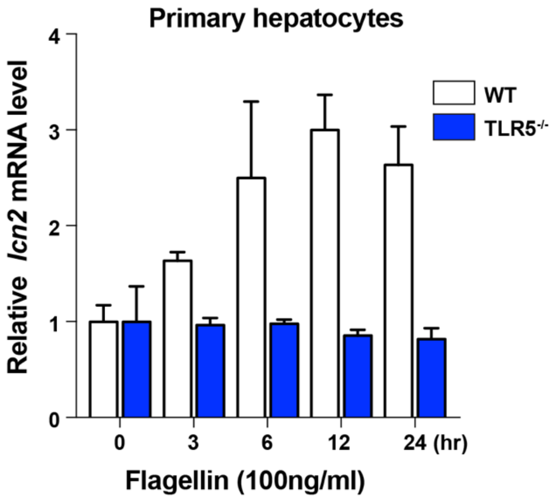

Supplemental Figure 2. Lcn2 expression in primary hepatocyte cultures of WT or TLR5$^{-/-}$ mice treated with flagellin. Hepatocytes were isolated from WT or TLR5$^{-/-}$ mice (n = 5) by collagenase perfusion (Noh et al. 2018). After allowing cells to adhere for 12 h, cultures were treated with flagellin (100 ng/ml). Lcn2 expression was measured by qPCR at the indicated times after treatment.

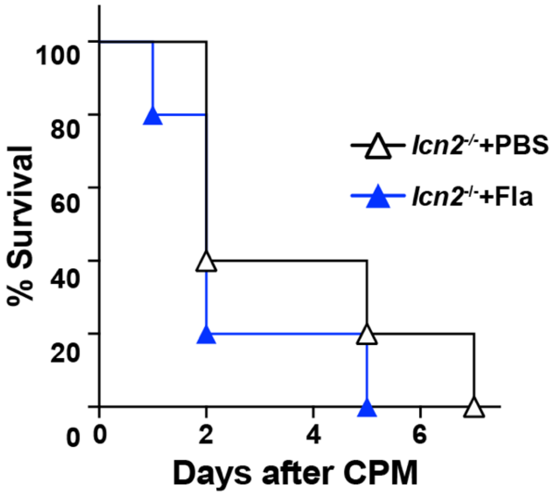

Supplemental Figure 3. Kaplan-Meier survival plot of *lcn2*$^{-/-}$ mice treated with flagellin or PBS 30 min prior to CPM treatment (n = 5 per group).

References

Noh JR, Kim YH, Kim DK, et al. (2018) Small heterodimer partner negatively regulates C-X-C motif chemokine ligand 2 in hepatocytesduring liver inflammation. Sci Rep 8: 15222)
